# A qualitative assessment of women’s perspectives and experience of female genital mutilation in Iraqi Kurdistan Region

**DOI:** 10.1186/s12905-019-0765-7

**Published:** 2019-05-16

**Authors:** Hamdia M. Ahmed, Sherzad A. Shabu, Nazar P. Shabila

**Affiliations:** 1 College of Nursing, Hawler Medical University, Erbil, Kurdistan Region Iraq; 2 Department of Community Medicine, Hawler Medical University, Erbil, Kurdistan Region Iraq

**Keywords:** Female genital mutilation, Tradition, Sexual desire, *haram* hands, Awareness, Erbil governorate

## Abstract

**Background:**

Female genital mutilation (FGM) is prevalent in Iraqi Kurdistan Region, but there is a lack of adequate knowledge about how the practice is perceived by the women population who are the direct victims of the practice. This study aimed to assess the knowledge, beliefs, and attitude of a sample of Kurdish women of FGM and identify the main enabling factors for performing this practice and the barriers to ending it.

**Methods:**

This qualitative study was based on six focus groups involving a sample of 51 women. We used a topic guide to lead discussions, which included questions on women’s perspectives of different aspects of FGM such as the reasons for practicing it, the positive and negative consequences, the continuation of the practice and tackling this problem in the community. Content analysis was used for the qualitative analysis of the data.

**Results:**

The women had poor knowledge about different aspects of FGM particularly concerning the procedure and the consequences. The mutilated participants revealed the devastating experience of the pain and the psychological effects they have experienced. Reducing sexual desire, having *halal* (permissible by *Allah*) hands, and religious requirement were the main reasons for practicing FGM. Reduction in women’s sexual desire and the related social problems with the husband were the main problems identified to be associated with FGM. Most women did not support the continuation of FGM practice, but some women still think that FGM should be left to the people’s preference. The participants identified raising people’s awareness, active involvement of religious leaders in prevention efforts and the issuance and enforcement of legislation against FGM as the primary measures to reduce FGM practice.

**Conclusion:**

Passing through FGM at childhood is an overwhelming experience with long-term effects for women. There is still a significant segment among the women population that do not oppose the continuations of FGM and need religious and scientific evidence against FGM. Some reasons for practicing FGM are deeply embedded in the culture and traditions, and there is a need for extensive efforts to raise the awareness of the population and change their thoughts and behavior about FGM.

**Electronic supplementary material:**

The online version of this article (10.1186/s12905-019-0765-7) contains supplementary material, which is available to authorized users.

## Background

Female genital mutilation (FGM) is any procedure involving partial or complete removal of the external genitalia of girls or women or any other injury to their genitalia for reasons other than medical indications [1]. FGM is divided into four main types in terms of the extent of the genital tissue cutting. Type I or clitoridectomy includes partial or total removal of the clitoris, while Type II or excision includes a partial or complete cut of the clitoris, labia minora and/or labia majora, and Type III or infibulation includes the narrowing of the vaginal orifice and creating a covering seal. Type IV FGM is any other harmful practice to the female genitalia, including pricking, piercing, or scraping [2]. FGM is a well-recognized violation of the rights of women and girls and is considered an extreme type of discrimination and inequality against girls and women. Since FGM is mainly performed for minors, it is considered to be a violation of the children’s rights. In addition to that, such a practice undermine the rights of these girls and women to health, security and physical integrity. Besides it exposes them to torture and inhuman treatment. As a consequence, the procedure might also result in death [1].

Health risks and consequences are not uncommon with FGM. Examples of some immediate consequences of FGM include pain, bleeding, infection, and difficulty in passing urine. Sometimes, it might result in chronic pain, chronic infections, poor quality of sexual life, birth complications, and psychological problems as long-term consequences [2–4]. Literature reveals that FGM is a tradition that is deeply rooted in many African countries and some Asian and Middle East countries [5]. All over the world, it is estimated that around 100–140 million women have experienced some types of FGM [6]. It is also estimated that each year three million girls below the age of 15 are at risk of this practice [5]. In the Middle East, FGM is commonly practiced in Egypt, Sudan, Yemen, and Iraq. The prevalence of FGM among 15–49 years girls and women in 2014–2015 was 87% in each of Egypt and Sudan, 19% in Yemen, and 8% in Iraq [7].

Although the prevalence of FGM in Iraq as a whole is relatively low compared to other countries where it is practiced, FGM is particularly common in the Iraqi Kurdistan Region. A prevalence of around 40% had been recorded for such a practice [6]. This prevalence varies by geographical locations. A prevalence of 4% was recorded in Duhok governorate, compared to 58% in Erbil governorate and almost 70% in some specific rural areas of Sulaimania governorate [8–10]. In general, the prevalence is noticeably lower among females aging below 20 years old (23%), which could indicate a decreasing trend of this practice. However, this rate remains considerably high and need to be dealt with as a significant health concern [11]. The most prevalent type of FGM in Iraqi Kurdistan context is Type I [8]. It is not clear why this particular type is common in the region. While there is some belief in the region that FGM is required, there is a general agreement that FGM should be limited to the mildest form [8]. While cutting and injuring types of FGM are known to be practiced in the region, infibulation or Type III is not known in the Kurdish culture.

The health consequences of FGM are becoming serious concerns. People’s awareness in Iraqi Kurdistan was considerably raised about the health concerns related to FGM through a series of awareness and advocacy campaigns. In 2007, a media and advocacy campaign against FGM was launched in Iraqi Kurdistan Region in response to media reports of a high prevalence of FGM [12]. In 2011, the domestic violence legislation was issued by the parliament of the Iraqi Kurdistan Region, which prohibited and criminalized FGM in Kurdistan [13]. The Kurdistan regional government established a higher council for women’s affairs and a general directorate to combat violence against women at the Ministry of Interior [8]. The awareness and attitude of the people and particularly women about FGM might have been affected by these efforts. However, no studies have examined the change in awareness about FGM as a result of this awareness campaign. Moreover, merely recognizing the harms of a practice that is deeply embedded in the roots of the society might not be adequate for totally eradicating this practice [14].

Regardless of the causative factors, women, especially little girls are the first victims of FGM, and their health-related problems indirectly affect the social, psychological and emotional health of families. Exploring the perspectives and experience of women themselves regarding FGM can give a clearer picture of the impact of this practice and help in finding effective approaches for its eradication. This information will also help in understanding the roots of the problem and enhancing the effectiveness of preventive programs. Limited research has examined how women think about this practice and what are the motivating factors for practicing FGM in the population.

This study aimed to assess the knowledge, beliefs, and attitude of a sample of Kurdish women of FGM and identify the main enabling factors for performing this practice and the barriers to ending it.

## Methods

### Setting

This qualitative study was conducted in Erbil governorate, the capital of Iraqi Kurdistan Region. Erbil governorate was chosen because it is the capital of the Kurdistan Region and include people from the different parts of Kurdistan. Moreover, Erbil governorate contains a diversity of districts and communities including those who commonly practice FGM and those who never practice it.

### Sampling

This qualitative study based on six recorded focus groups was conducted in Erbil governorate, Iraqi Kurdistan Region, from July to October 2016. A total of 51 women were included in this study. The participants were selected from different settings to represent various groups of Erbil women populations. A total of 17 women were selected from the women accompanying patients attending Erbil Maternity Hospital, and they were involved in two focus groups. The third focus group involved a sample of eight women from the employees of a governmental office in Erbil city to represent highly educated women. Three other focus groups were conducted in three sub-districts around Erbil city; Banslawa, Kasnazan, and Baharka. These three sub-districts were purposively selected to represent people of different geographical origin and with different views and practice of FGM. A purposive sample of women visiting the main primary health care center in each of these three sub-districts was invited to participate in the focus groups. In total 51 women participated in the six focus groups with each focus group involving eight to nine participants. The number of focus groups was determined by data saturation as we decided to limit the focus groups to six when adequate data saturation was reached as no new information and themes were emerging from the data. Thus the number of participants was limited to 51.

Participants were selected by means of purposive sampling based on a profile comprising a set of selection criteria including girls and women aged 18 years and above, not severely ill, able to communicate in any of the two Kurdish dialects of Sorani and Badini, comfortable to talk about the potentially sensitive topic of FGM and able to provide informed consent to participate in the focus groups. Besides the selection criteria, we aimed to obtain a diverse range of participants in terms of age, marital status, educational level, socioeconomic level, and experience with FGM and geographical origin within the Iraqi Kurdistan Region. Recruitment was coordinated with the administration at the workplace or the health settings. Moreover, we tried to snowball via the networks of the selected participants to recruit other participants with similar characteristics such as being mutilated and availability to talk about this sensitive topic in focus groups.

### Data collection

The six focus groups were facilitated between July and October 2016. The venue of the focus groups was the meeting room in the selected settings. Two researchers, a moderator and an observer facilitated each focus group. The researchers attended a one-day training workshop, which consisted of short, interactive presentations, group sessions, reflective work, and a practice interview. The workshop ended with a discussion of experiences encountered during their practice interview, including problems, pitfalls, and suggestions.

At the beginning of each focus group, the aim of the study and the main rules regarding recording the discussions and anonymity of the participants were explained to the participants. Signed informed consent was obtained from each participant after explaining the aim of the study and the main rules regarding recording the discussions and anonymity of the participants and before starting the focus groups. Verbal consent was obtained from the illiterate participants after the interviewer had read out the informed consent statement to them. Each focus group lasted approximately sixty minutes. The study protocol was approved by the Research Ethics Committee of Hawler Medical University.

A topic guide was used to lead focus groups. It included questions on women’s perspectives around different aspects of FGM such as understanding of FGM, reasons for practicing it, the positive and negative consequences, attitude toward continuation or discontinuous of the practice and suggestions for tackling this problem in the community (Additional file 1). The participants were also asked to describe their own experience with the practice. Each session was concluded when no new information was emerging from the discussion. A debriefing discussion was conducted between the moderator and the observer at the end of each focus group.

All the discussions were conducted in Kurdish language and were recorded in full. The audio recordings of the discussions were transcribed and translated into English. Verification of the translation was subsequently carried out by an additional native Kurdish speaker fluent in English.

### Data analysis

Content analysis was used for the qualitative analysis of the translated transcripts. The transcripts were reviewed by two researchers independently. The condensed meaning units were identified and were labeled with codes. Emerging coding was used to obtain categories. These categories were used to identify and formulate themes and sub-themes. A greater emphasis was placed on themes and sub-themes repeated by more than one group, initially raised themes and sub-themes, strong feelings, or themes and sub-themes of long discussions. The discordant views were included to highlight differing experiences or perceptions of individuals and groups.

## Results

The age ± SD of the participants was 36.3 ± 10 years. Most of the participants were 30–44 year old (45.1%), illiterate (37.3%), married (74.5%), from outside Erbil city (66.7%), not employed (72.5%) and had experienced FGM (56.9%). FGM was more common among the older aged, poorly educated, married, and unemployed participants in addition to those from Erbil city.

The details of the sociodemographic characteristics and the FGM status of the study participants are shown in Table 1.

**Table 1:** Sociodemographic characteristics and the FGM status of the study participants

Characteristic	Mutilated		Not mutilated		Total	
	No.	%	No.	%	No.	%
Age (years)						
18–29	6	35.3	11	64.7	17	33.3
30–44	15	65.2	8	34.8	23	45.1
≥ 45	8	72.7	3	27.3	11	21.6
Education level						
Illiterate	12	63.2	7	36.8	19	37.2
Primary school	8	100	0	0	8	15.7
Secondary school	5	50	5	50	10	19.6
College	4	28.6	10	71.4	14	27.5
Marital status						
Married	23	60.5	15	39.5	38	74.5
Single	6	46.2	7	53.8	13	25.5
Residence						
Erbil city	13	76.5	4	23.5	17	33.3
Outside Erbil city	16	47.1	18	52.9	34	66.7
Employment status						
Employed	5	35.7	9	64.3	14	27.5
Not employed	24	64.9	13	35.1	37	72.5
Total	29	56.9	22	43.1	51	100

### Knowledge about FGM

A group of participants indicated that they have not heard about FGM or they have only heard about it from the news and TV. This pattern of knowledge was particularly common among the well-educated and non-mutilated participants. Another group, primarily the mutilated and both well and poorly educated, indicated that FGM is a traditional practice in the Kurdistan Region that was more prevalent in the past.

*“We do not have the circumcision for women in our family at all. I did not know about it until I became an adult. I heard about that on TV. They say it is a tradition.” (A participant from group 2).*

*“I do not know much about it and have no idea what is it exactly. I only heard that it is a bad traditional practice.” (A participant from group 5).*

### Procedure and performers

All the participants who had some knowledge about FGM indicated that traditional birth attendants, locally called *maman*, or elderly women practice FGM and medical people are not involved or even not needed. They agreed that FGM is practiced in secret.

*“Elderly women perform FGM. There is no need for doctors. Sometimes traditional birth attendants do it and use tomato paste on the wound which gives a burning sensation. They cut the head of it.” (A participant from group 4).*

*“In the past, there were no female doctors. They were filling a basin with water and put the girls in it for circumcision.”(A participant from group 3).*

Some participants stated that a small part of female genitalia is cut during FGM while others indicated that a large part is cut or even there is a complete cut. This knowledge was particularly more common among the well-educated and mutilated groups of participants.

*“Some cut a large piece, and others cut only a small piece.” (A participant from group 6).*

*“I think they usually cut a small piece of it, not all of it.” (A participant from group 3).*

*“Until now I do not know what they do and what they cut.” (A participant from group 2).*

The participants, particularly the mutilated ones, indicated that the mother or grandmother usually decides to do FGM to the girls, while the fathers or men are not involved in such decision making.

*“The mothers or grandmothers are usually the ones who decide to practice FGM to their daughter rather than the father.” (A participant from group 4).*

### Experience of FGM

The mutilated participants talked about their own experience of going through FGM at a very young age, while some non-mutilated participants talked about or hearing about the practice from family and friends. They emphasized the pain and the psychological effect of going through such a devastating experience at such a young age. However, some participants indicated that they do not remember much about their experience since they were mutilated at a very young age. Many participants, particularly the non-mutilated and poorly educated from outside the city, indicated that FGM is no longer practiced in the Kurdistan Region.

*“I was just a kid when I was circumcised, and I did not know what that was.” (A participant from group 2).*

*“I was circumcised when I was a child, and till now I am afraid of it. The fear has remained with me.” (A participant from group 3).*

*“I was in my first year at the primary school when I was circumcised, and till now I remember it. I will never forget that day.” (A participant from group 3).*

*“I remember when I was circumcised. I had bleeding. I have not circumcised my daughters.” (A participant from group 6).*

*“I have not undergone circumcision. I wanted to do it, and I went to a traditional birth attendant who said that I do not do it any longer.” (A participant from group 4).*

*“I have relatives who have undergone circumcision and say that they wish that they not have done it.” (A participant from group 6).*

*“I have not done it and had no idea about it. I know our neighbor who has done it.”(A participant from group 6).*

### Reasons for practicing FGM

Regarding the reasons for practicing FGM, many participants, particularly the educated and non-mutilated from the city center, indicated that they do not know why FGM is practiced.

*“I still want to know what the reason is. I would like to know why the people do that to girls.” (A participant from group 2).*

*“We do not know why they do it and what will change if they cut this part or not.” (A participant from group 3).*

The other participants provided three main reasons for practicing FGM; reduce sexual desire, have *halal* hands and religious requirement. The participants pointed out that people think that FGM reduces sexual desire in girls and women and thus protect them from engagement in premarital sex or sexual promiscuity. This viewpoint was primarily emphasized by the poorly educated mutilated and non-mutilated participants from outside the city.

*“We heard people saying by doing circumcision females will not do the wrong things, as circumcision decreases sexual desire. However, women will pay for the consequences of that.”(A participant from group 2).*

*“People say circumcision decreases the sexual desire of girl as she needs to be shy and not to go outside the home.” (A participant from group 2).*

*“A married woman said once I wish I have done it to avoid doing the wrong things.” (A participant from group 4).*

*“If the husband is at work or far from home, the wife might not be able to control her sexual desire. Therefore, it is better to be done. If we do it, the woman can control her sexual desire.” (A participant from group 4).*

*“The circumcised woman will be somehow shy compared to other non-circumcised women.” (A participant from group 1).*

The participants indicated that some people consider uncircumcised girls and women have *haram* hands, and thus the food they prepare or offer is *haram* (forbidden by *Allah*). People also think that women with no FGM are considered dirty. Some people even think that if anything is touched or done by an uncircumcised girl and women will become *haram,* and nobody should eat or drink it. Thus the uncircumcised girl and women will become stigmatized and isolated in the family and community. Such social stigma will push the family to subject their daughters to FGM and even push adult females to pass through FGM at a later stage. This perspective was emphasized by the non-educated and mutilated participants from outside the city.

*“We heard religious leaders saying if we do it our hands will be halal, but this is what they fabricate.” (A participant from group 2).*

*“Before they used to say that one should not drink water from a woman’s hand who has not undergone circumcision, as it is haram, but now it is normal.” (A participant from group 3).*

*“If you do not do it, according to religion your hand will be haram.” (A participant from group 4).*

*“People say that it is haram when a woman does not do it and my mother used to say that it is not good if a woman does not do a circumcision.” (A participant from group 2).*

*“The woman who is not circumcised, cannot kill an animal like a chicken.” (A participant from group 1).*

*“A woman who was not circumcised complained that her husband always insults her and tell her bad things such as you are dirty.” (A participant from group 2).*

The participants, notably the non-educated and mutilated, stressed that many people believe that FGM is a religious requirement and a *Sunnah* (the tradition of the Prophet Muhammad) and should be practiced by all Muslims.

*“People were doing circumcision before as it was considered a Sunnah.” (A participant from group 3).*

Other reasons for practicing FGM included to become shy and get lucky that were primarily emphasized by the educated and non-mutilated participants from the city.

*“I do not know. People say that if women are not circumcised, they do not become lucky in life.”(A participant from group 3).*

### Consequences of FGM

Some participants including some who have experienced FGM indicated that FGM would not cause any problem. However, many of them stressed that the practice has no any benefits even if it does not cause problems.

*“I have undergone circumcision, and have no any problem with that.” (A participant from group 1).*

*“It is meaningless. It has no benefits. So why to do it.” (A participant from group 3).*

*“It has no any benefits neither from the scientific nor the sexual aspect.” (A participant from group 3).*

*“If there is evidence that it is scientifically sound, let them prove it. I think it should be done only for boys.” (A participant from group 2).*

*“We do not know the advantages of circumcision, but we know it has some adverse effects.” (A participant from group 2).*

Most participants, especially the educated and poorly educated mutilated, indicated that FGM could cause complications such as bleeding and pain and long-term effects such as psychological problems and reduced sexual desire. The main focus of the discussion was about the problem of reduced sexual desire and pleasure in mutilated women and the associated marital and social problems that might result in divorce or the husband marrying another woman or engage in an extramarital relationship to get sexual satisfaction.

*“I heard about one case of a woman who has done circumcision, and she had a bad relationship with her husband that resulted in divorce due to the circumcision.” (A participant from group 5).*

*“The problems of divorce and men marrying more than one woman are increasing due to women circumcision.” (A participant from group 6).*

*“Those who do not do circumcision are better. Those who do circumcision do not get any benefit from it. Circumcision in women causes bleeding and decreases sexual desire.” (A participant from group 3).*

*“I have no sexual desire. This, of course, creates problems with my husband.” (A participant from group 6).*

*“A woman told me that she does not get any sexual pleasure with her husband. They have cut it all. She says that it was better for her not to get married.” (A participant from group 3).*

*“If circumcision is not done, women will not suffer from decreased sexual desire so men will not go outside and engage in wrong relations with other women.” (A participant from group 1).*

*“Circumcision in women decreases the sexual desire of women especially if there is too much cut. This creates problems between the woman and her husband.”(A participant from group 5).*

*“My husband always makes fun of me by saying you are circumcised. I have sexual problems with him, and I always tell him it is not my guilt.” (A participant from group 2).*

The mutilated participants, especially the educated ones, emphasized the terrible experience of going through FGM at a very young age, and the psychological effect and the fear and stigma resulted from such experience that remained with them until now.

*“Doing circumcision will hurt our feeling especially when we do it at 13-14 years old.” (A participant from group 4).*

*“Circumcision affects us psychologically, and we suffer from fear, and this will remain with you every time you talk about it.” (A participant from group 4).*

The study participants also highlighted the heartless or non-human aspect of subjecting the young girls to the terrible experience of FGM without their consent.

*“Doing circumcision for these very young girls is heartless.” (A participant from group 3).*

### Continuation of FGM

Most participants indicated that it is better not to practice FGM. They thought that not practicing it will not be against the religion as it is not mentioned in religious scripts. They provided examples of local *mullas* saying there is no bad doing in not practicing it. Some participants even mentioned that FGM should not be practiced even if the local *mullas* advise to do it. This perspective was particularly common among educated and non-mutilated participants.

*“I think, there will be no problem with not doing circumcision for women. We can still kill a chicken. Even if the mullah says it should be done, there is no problem with not doing it.” (A participant from group 1).*

*“Better not to do circumcision for women. Every time we talk about circumcision, girls get scared. Religion has not talked about it.” (A participant from group 4).*

*“Women circumcision is meaningless. Why should we do it? The mullah said even in the Prophet’s time some people did it and others did not. Currently, people are doing it less frequently than before.” (A participant from group 4).*

Some other participants, particularly the mutilated and non-educated from outside the city, preferred to leave the practice of FGM to the people themselves to decide whether to practice it to their daughters or not.

*“It should be up to the people themselves whether they like to do circumcision to girls or not.” (A participant from group 6).*

Most of the participants indicated that they have not done FGM to their daughter and will not do it for them. Some participants indicated that they had to subject their daughters to FGM due to their belief in this tradition or due to the family and social pressure. The latter was primarily emphasized by the mutilated, non-educated participants from outside the city.

*“I have undergone circumcision, but I will never do it for my daughters.” (A participant from group 1).*

*“Recently, people stopped doing circumcision for girls. My daughter is nine years old and is not circumcised.” (A participant from group 2).*

*“I have three daughters, and I have not even thought about doing circumcision to them.” (A participant from group 6).*

*“I will never circumcise my daughters.” (A participant from group 2).*

*“I have undergone circumcision, and it is not good. I have two daughters who have also undergone circumcision.”(A participant from group 6).*

### Measure to prevent FGM

The participants pointed out that the people are not much aware of the different aspects of FGM including the reasons for practicing it and the associated complications of the procedure. They stressed the importance of increasing the awareness of the people about the adverse effects and unnecessity of practicing FGM from the religious and health aspects.

*“There is a need for organizing awareness courses for women and advise them not to do the circumcision. There should be punishment for those who do it.” (A participant from group 4).*

*“We need to gather mothers at schools since girls do not understand anything about that.” (A participant from group 2).*

The participants also emphasized the vital role of religious leaders in preventing FGM. They thought that religious leaders need to talk to people about FGM in *khutba* (public preaching) and media. They stressed that religious leaders should not feel ashamed to mention this topic in their preaching. They indicated that being Kurds and lacking knowledge about the Arabic language, people might have misunderstood or misinterpreted religious scripts. Therefore, religious leaders should have an essential role in explaining these issues to the people. This viewpoint was more common among the non-educated and mutilated participants from outside the city.

*“The mullahs should talk about female circumcision during khutba and tell people not to do it.” (A participant from group 4).*

*“We are all Muslims and listen to the mullah. They should come on the TV and say do not do it and this will have its impact.” (A participant from group 4).*

*“The mullah should talk to people about female circumcision. The people of Erbil listen to mullah more than anybody else like doctors.” (A participant from group 6).*

*“Sometimes people blame the mullah when he talks about these issues and says that he should feel ashamed to talk about such issues on the TV.” (A participant from group 4).*

*“The Arab people will read the Holy Quran and understand it, but in our case [Kurds], we understand it according to how it is translated or explained for us.” (A participant from group 4).*

Some participants, particularly the educated, non-mutilated from the city, stressed the importance of prohibiting FGM by law and enforcing the law by punishing those who practice FGM or subject girls to the practice.

*“I very much prefer to have a law in order to prohibit female circumcision officially.” (A participant from group 3).*

*“There should be a law from the Parliament. Otherwise, it will be just talks. The disadvantages of circumcision should be clarified scientifically.” (A participant from group 2).*

*“There should be a law and disciplinary measures to be taken with those who do circumcision to girls. They should check if it is mentioned in the Holy Quran or Sunnah.” (A participant from group 3).*

*“I prefer to punish those who do circumcision to their girls. What is the guilt of a 5-year old child to cut this piece of tissue from her body?” (A participant from group 3).*

Some participants emphasized the importance of having clear scientific and *sharia* evidence about FGM before prohibiting it. This viewpoint was primarily emphasized by group 1 participants, which included mainly poorly educated and mutilated women from outside of Erbil city.

*“We need to have both scientific and Shari’a evidence to decide on doing or not doing female circumcision.” (A participant from group 1).*

*“You can conduct scientific research and consider their results. Whether to prohibit or support female circumcision, we need to have good evidence.” (A participant from group 1).*

## Discussion

The aim of this study was not to determine the prevalence of FGM and thus the sample was not random or representative. However, having 56.9% of the participants experienced FGM supports previous studies from the region that have identified FGM as a continuing, important problem in the Iraqi Kurdistan Region [8, 9]. The participants emphasized the terrible experience of going through FGM and the pain and the psychological effect of such experience. Going through the devastating experience of FGM or observing the practice at such a young age results in significant fear and other psychological effects in addition to the immediate consequences of pain and bleeding [15].

This study showed that traditional birth attendants or elderly women perform FGM with no involvement of the health professionals. Other studies from Iraqi Kurdistan Region also revealed that FGM is performed by non-professional old ladies or traditional circumcisers with no or limited involvement of health professionals. Traditional circumcisers primarily perform FGM in most countries where FGM is performed [16, 17]. In a few countries such as Egypt, health professionals are primarily responsible for performing FGM [18]. These old ladies and traditional circumcisers use very basic tools such as a razor blade and perform the procedure under unhygienic conditions. Therefore, these victims are at a high risk of developing complications, including bleeding and infection [15]. However, this should not make a case for the medicalization of FGM as it encourages its legalization.

The study participants did not have any clear idea of what part and how much of the female genitalia is cut during FGM. Previous research has shown that type I FGM or clitoridectomy is the most common type practiced in the Iraqi Kurdistan Region (76–99%) [8, 9, 19]. Lack of women’s knowledge about the types of FGM had been reported in other countries such as Sudan, Nigeria, and Egypt [20]. Lack of knowledge about what FGM involves exactly might contribute to the increased prevalence. If women know that the procedure involves removing or damaging important parts of the genitalia, they might be restrained from practicing it for their daughters.

This study revealed that the mothers or grandmothers usually decide to do FGM to the girls, a finding that agrees with other studies from Iraqi Kurdistan Region where the fathers and the men members of the family are usually not involved in taking the decision [15]. In most other settings such as East Ethiopia, mothers and grandmothers play a dominant role in deciding and organizing FGM for their daughters [21]. It is thought that mothers want to optimize their daughters’ prospects, and they do have a fear of violating the tradition, which pushes them to subject their daughter to the practice. This can also be considered a form of internalized oppression among women. As women have been targeted, abused, or oppressed over a long period of time, they might have internalized or believed the myths and misinformation that society has communicated to them about FGM. Women might have internalized the stereotype that they are not clean or the food from their hands is not halal if they are not circumcised. There is a need for the development and refinement of methods for assisting the women to recover from the effects of such oppression [22]. Therefore, preventive efforts should primarily involve educating, raising the awareness and changing the behavior of the mothers.

The three main reasons provided by the study participants for FGM practice included reducing sexual desire in girls and women, having *halal* hands and religious requirement. Another study from Iraqi Kurdistan Region also identified religious obligation and making the food handled by girls and women *halal* as the main reasons for practicing FGM [15]. Religious obligation is an essential reason for practicing FGM in some other countries, including Iran (2.8%) [17], Sudan (26%) [20], and Nigeria (10%) [23]. No religious scripts advise or encourage FGM, but many people believe that FGM is supported by religion [24]. However, Islamic scholars have different positions concerning FGM. Some religious leaders encourage FGM, others consider FGM unrelated to religion, while some scholars work on eliminating the practice [1, 25]. In the Iraqi Kurdistan Region, the religious requirement remains an important reason for subjecting girls and women to FGM [8, 9]. A number of well-known scholars in the region have condemned FGM in public and denied any connection between Islam and FGM, while others remained silent or even encouraged the practice [19, 24]. It is essential to examine the viewpoints of religious leaders in the region to determine the reasons for having different positions about FGM and find out whether their specific positions are related to purely religious or other cultural factors [15].

As this study revealed, uncircumcised women in Iraqi Kurdistan Region are sometimes considered dirty and have *haram* hands, and surprisingly some people even do not eat or drink from their hands if they are not circumcised [15, 26]. These uncovered aspects of FGM can contribute significantly to having a high prevalence of FGM in the region.

Social or cultural tradition is the primary reason for practicing FGM in Iraqi Kurdistan Region [8, 9]. It is also an important reason for practicing FGM in many other settings, which sometimes surpass religious requirement as the most common reason [18, 27]. In many other settings where FGM is prevalent, female genitals are commonly seen as dirty, and FGM is practiced for a child for being made clean [28]. However, the issue of *halal* hands in very specific to the Iraqi Kurdistan Region that requires further investigations and changing such beliefs might play an essential role in stopping FGM practice in the region.

Many problems related to FGM were identified by the study participants with the primary emphasis on the reduced sexual desire in women and subsequent marital problems. In another study from Iraqi Kurdistan Region, women identified loss of sexual desire and/or arousal as the primary complications of FGM with no or little focus on the other immediate or long-term physical or psychological consequences of FGM [15]. The participants’ primary focus on the sexual adverse effects of FGM could be related to the excessive damage to the female genital organ produced by non-professional traditional circumcisers. Unfortunately, limited research has assessed the complications of FGM in Iraqi Kurdistan Region because FGM is mainly practiced in secret. This specific focus on the sexual adverse effects might be because some of the study participants who have experienced FGM were already suffering these complications. Linking FGM with ensuring premarital virginity and marital fidelity and cultural obsession about female chastity and virginity in the conservative societies such as Iraqi Kurdistan Region might have directed the discussions towards these complications in particular. Research from Eastern Sudan has shown that mutilated women have a high awareness of decreased sexual desire and pleasure, lack knowledge of the other complications [20].

The immediate consequences of FGM such as pain, bleeding, and infection frequently occur very soon after the procedure with the victim is still a child. This fact could be the reason for some women to claim that FGM does not cause any problems or for the lack of participants’ emphasis on immediate complications. Poor knowledge about the serious complications could be attributed to having type I FGM as the most prevalent type of FGM practiced in Iraqi Kurdistan Region, which could be less risky than the other types of FGM. For example, another study from Erbil city showed that 99.6% of cases had type I FGM, and only 6.3% of the victims reported the occurrence of complications [8]. In other settings, research has identified bleeding, genital tissue swelling, and urine retention as the main immediate complications [2] and urinary tract infections, bacterial vaginosis, dyspareunia, and difficult labor as the main long-term complications [29].

Most participants believed that it is better not to practice FGM, while others favored to leave it to the people to decide to do it or not. Other recent studies have revealed that the continuation of FGM is not supported by most women in Iraqi Kurdistan Region as they think that it is not associated with any benefits for the women [15, 26]. However, two old studies from 2008 reported high support of the Kurdish women to the continuation of FGM practice particularly the mutilated participants (36.6 and 28%) [8, 9]. This might indicate that there is less support for the continuation of FGM in the region with the pass or time. However, FGM is still prevalent in the region even if most of the people are against it. Presence of such contradiction calls for having a more in-depth exploration of this issue. The support of the girls and women to the continuation of FGM is also high in other countries. Low economic status, poor education, and poor exposure to media were associated with a higher level of support to FGM [30]. Therefore, increasing media coverage, awareness and education, and poverty reduction are important for changing women’s attitudes toward discontinuation of FGM.

Most of the participants stressed that they have not done FGM to their daughter and will not do it for them. However, some participants revealed that they had practiced FGM to their daughters due to their belief in this tradition or due to social pressure. Another study from the Iraqi Kurdistan Region revealed that the circumcised mothers in addition to the mothers’ education level, employment status and the household wealth index were significantly associated with doing FGM to the daughters [31]. A study from Iran revealed that attitude and subjective norms are the strongest predictors of mothers’ intentions to practice FGM to daughters. Old age, poor education, and rural area residence were significantly associated with having positive attitudes among mothers toward FGM and feeling more social pressure to allow FGM [32]. Therefore, intervention programs to decrease FGM need to focus on changing mothers’ neutral or positive feelings toward FGM into negative attitudes and easing the observed social pressure that pushes them to mutilate their own daughters.

The participants emphasized the need to increase the awareness of the people, the involvement of religious leaders and prohibiting FGM by law to reduce FGM in Kurdistan Region. The need to raise the awareness of the people in preventing FGM was also shown in previous studies [15]. With the advocacy and awareness campaigns in the region during the last decade, more people have become aware of the complications of FGM. Nevertheless, rejecting a traditional practice that is deeply rooted in the society cannot be achieved merely by understanding its harms. Knowledge alone does not necessarily affect health behavior change particularly in an active manner [14]. The participants also stressed the importance involving religious leaders in the efforts to reduce the prevalence of FGM in Kurdistan Region. The need to actively engage religious leaders in the FGM problem was also revealed in previous studies [15]. Religious leaders might be able to influence the shaping of people’s behavior. Such influence can be particularly important in FGM as the practice is commonly perceived as a religious requirement. Religious leaders can have a crucial influence on the discontinuation of FGM [33]. Some programs have shown that if communities decide to abandon FGM with the help of religious leaders, rapid elimination of FGM can be attained [34, 35].

The participants also emphasized the need to prohibit FGM by law. Legislations that prohibit and/or criminalize FGM have been issued in many countries where FGM is prevalent including Iraqi Kurdistan Region. However, enforcement of these legislations is still a concern, and their effects on reducing FGM prevalence are largely understudied [36–38]. Other studies have shown that the people in Kurdistan have inadequate knowledge and awareness about the presence of legislation prohibiting FGM in Iraqi Kurdistan Region and the details of such legislation [15], which calls for raising the awareness of the people about the existing laws.

Some study participants asked for having clear scientific and Sharia evidence about FGM before prohibiting it. Another study from the Iraqi Kurdistan Region recognized the importance of providing a clear message to the religious leaders in the region with clear evidence about FGM to convey it to the people [19]. Such a message should be provided by local scholars and the concerned authorities including the Ministry of Endowments and Religious Affairs. The+re is also a need to produce clear evidence about the health and sexuality consequences of FGM [39]. It is essential to have this evidence produced locally by reputed local scholars so that the people including the religious leaders can use the proof in the efforts of banning the practice.

### Strengths and limitations

Using a qualitative design in this study helped us to explore the perspectives of both mutilated and non-mutilated women about different aspects of FGM in an in-depth manner. Using focus groups and involving participants with different sociodemographic characteristics have encouraged having more interactive discussions and exploring various aspects of FGM problem. However, some participants might not have been able to express their experience and feelings in such heterogeneous groups and on such a sensitive topic. Although the focus group environment was open and friendly and the participants were willing to share experience and perspectives comfortably and freely, underestimation cannot be ruled out. Presence of well-educated and non-mutilated women in the sample might have restrained the other participants from talking comfortably. This study is also limited to participants from Erbil governorate only. Women from Duhok governorate where the FGM is not common or from Sulaimania governorate where FGM is more widely practiced might have different experience or perspectives. This study is also potentially limited by response bias as the focus group moderators were health professionals, who are known in the region to oppose FGM. Some study participants might have answered the questions in a manner that would be viewed favorably by the moderators. The study participants who were aware of the existence of the legislation criminalizing FGM in the Iraqi Kurdistan Region might not have expressed their potential supportive attitude toward FGM.

## Conclusions

There is relatively poor knowledge about different aspects of FGM among women particularly concerning the procedure and the consequences. Reduction in women’s sexual desire and the related social problems with the husband were the main problems identified to be associated with FGM. Passing through FGM at childhood is an overwhelming experience with long-term effects for women. There is still a significant segment among the women population that do not oppose the continuations of FGM and need religious and scientific evidence against FGM. Some reasons for practicing FGM are deeply embedded in the culture and traditions, and there is a need for extensive efforts to raise the awareness of the population and change their thoughts and behavior about FGM. Educating and raising awareness of the population, particularly the women, might play a key role in reducing FGM practice in Iraqi Kurdistan Region. Further research is needed to better understand the roots and motives to practice FGM in the region and to examine the role of different preventive measures in reducing the prevalence of FGM.

## Additional file

Additional file 1: Questions about knowledge, attitude, and practice included in the focus groups topic guide. (DOCX 17 kb)

## Abbreviations

FGM: Female genital mutilation
