# Supplementary material for: A qualitative assessment of women’s perspectives and experience of female genital mutilation in Iraqi Kurdistan Region
Source: BMC Womens Health. 2019 May 16;19:66. doi: 10.1186/s12905-019-0765-7 (PMC6521410; doi:10.1186/s12905-019-0765-7)
Supplement: Supplementary file 1 — Questions about knowledge, attitude, and practice included in the focus groups topic guide. (DOCX 17 kb) [file 12905_2019_765_MOESM1_ESM.docx]

**Additional file 1** Questions about knowledge, attitude, and practice included in the focus groups topic guide

| **Knowledge** | **Attitude** | **Practice** |
| --- | --- | --- |
| What is FGM?  What are the types of FGM?  Who decides on practicing it?  Who performs it?  Reasons for practicing FGM  (religious, maturity, sexuality, cleanliness)  Benefits of FGM  Health consequences of FGM  (pain, health problems, psychological upset) | What is your perspective regarding the continuation/discontinuation of FGM?  - Probes about:  Support, why?  Rejection, why?  What are the reasons behind the motivation for the continuation or discontinuation of the practice  Continuation:   - Religious requirement - Social environment (cultural traditions, fear of social stigma) - Promote cleanliness, women maturity, ensure virginity, prevent promiscuity,   Discontinuation   - Health problems - Painful experience - Erode females’ sexual feelings - Social environment (legislation, prosecution)   What factors are considered detrimental for the continuation or discontinuation of FGM   - Legislation and enforcement - Population awareness - Religious leaders' support - Civil society role   Do you have intentions to circumcise your girls?   - Ask why in both cases of Yes and No answers. | What is your experience with FGM?  (Personal experience, the experience of a family member or girls/women you know)  - Who decided  - Who performed  - What type of FGM  - At what age  - Why it was performed (religious, maturity, sexuality, cleanliness)  - Complications (Pain, health problems, psychological upset)  - Performed in secrete or not  - How the family perceived it? (considered something proud of)  Have you circumcised your daughters?   - Ask why in both cases of Yes and No answers. |
